# Clinical features suggesting renal hypouricemia as the cause of acute kidney injury: a case report and review of the literature

**DOI:** 10.1007/s40620-022-01494-8

**Published:** 2022-11-23

**Authors:** Tommaso Mazzierli, Luigi Cirillo, Viviana Palazzo, Fiammetta Ravaglia, Francesca Becherucci

**Affiliations:** 1 Department of Biomedical, Experimental and Clinical Sciences “Mario Serio”, University of Florence, Florence, Italy; 2 Nephrology and Dialysis Unit, Meyer Children’s Hospital IRCCS, Florence, Italy; 3 Medical Genetics Unit, Meyer Children’s Hospital IRCCS, Florence, Italy; 4 Nephrology and Dialysis Unit, Santo Stefano Hospital, Prato, Italy

**Keywords:** Renal hypouricemia, AKI, EIAKI, Uric acid

## Abstract

Hypouricemia is defined as a level of serum uric acid below 2 mg/dl. Renal hypouricemia is related to genetic defects of the uric acid tubular transporters urate transporter 1 and glucose transporter 9. Patients with renal hypouricemia can be completely asymptomatic or can develop uric acid kidney stones or acute kidney injury, particularly after exercise. Renal hypouricemia is especially challenging to diagnose in patients with acute kidney injury, due to the nonspecific clinical, hematochemical and histological features. No common features are reported in the literature that could help clinicians identify renal hypouricemia-acute kidney injury. Currently available guidelines on diagnosis and management of renal hypouricemia provide limited support in defining clues for the differential diagnosis of renal hypouricemia, which is usually suspected when hypouricemia is found in asymptomatic patients. In this paper we report a case of renal hypouricemia-acute kidney injury developing after exercise. We carried out a review of the literature spanning from the first clinical description of renal hypouricemia in 1974 until 2022. We selected a series of clinical features suggesting a diagnosis of renal hypouricemia-acute kidney injury. This may help clinicians to suspect renal hypouricemia in patients with acute kidney injury and to avoid invasive, costly and inconclusive exams such as renal biopsy. Considering the excellent outcome of the patients reported in the literature, we suggest a “wait-and-see” approach with supportive therapy and confirmation of the disease via genetic testing.

**Supplementary Information:**

The online version contains supplementary material available at 10.1007/s40620-022-01494-8.

## Introduction

Primary renal hypouricemia (RHUC) is caused by pathogenic variants in *SLC22A12*, encoding for the apical urate transporter 1 (URAT1; type 1 RHUC), or *SLC2A9*, encoding the basolateral glucose transporter 9 (GLUT9; type 2 RHUC). They are rare diseases with an autosomal recessive pattern of inheritance [1], although autosomal dominant inheritance is reported for either gene [2, 3]. RHUC may be completely asymptomatic lifelong. However, the clinical course can be complicated by uric acid (UA) kidney stones or acute kidney injury (AKI), usually occurring after exercise. Although representing the most frequent clinical manifestation of RHUC, RHUC-AKI can be clinically challenging to recognize, since laboratory testing and kidney biopsy findings are nonspecific. Consequently, RHUC is frequently suspected long after AKI, when blood tests show extremely low uric acid levels [4, 5].

We report the case of a young man affected by RHUC who developed AKI as the first disease manifestation, and we provide a review of currently available literature looking for clinical features suggestive of this diagnosis in patients with AKI.

## Case report

**Fig. 1:**
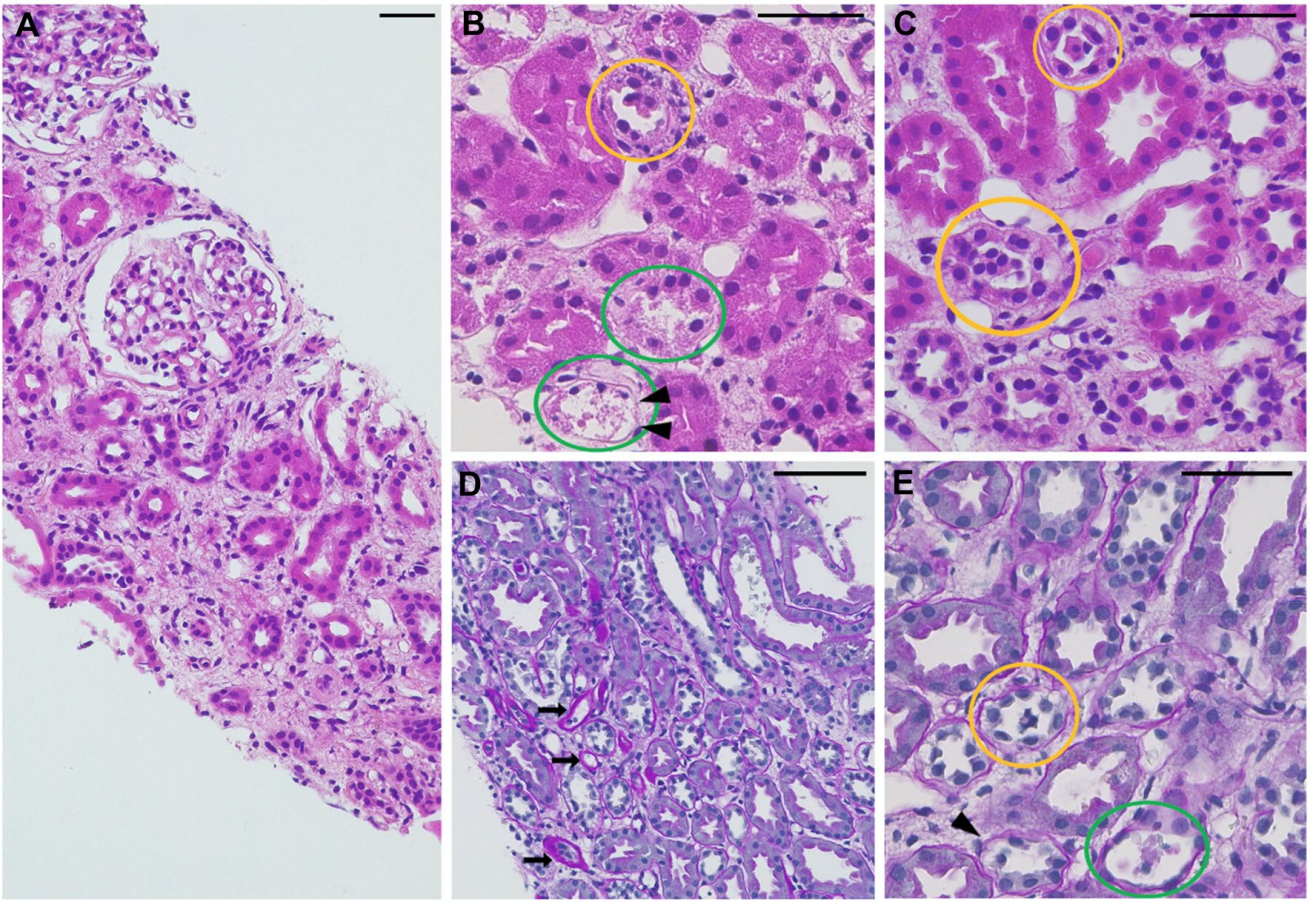
Kidney biopsy findings. **A** At lower magnification glomeruli appear unremarkable, while interstitial edema is present. **B**–**E** At higher magnification signs of acute tubular injury are present, with areas of tubular basement membrane denudation (arrowheads) and atrophy (arrows), presence of detached proximal tubular cells in tubular lumina (yellow circles) and coagulative necrosis of tubular epithelial cells with cell debris in the tubular lumina (green circles). (**A**–**C** hematoxylin eosin stain, **D** and **E** Periodic Acid Schiff stain, bars = 50 µm)

**Fig. 2:**
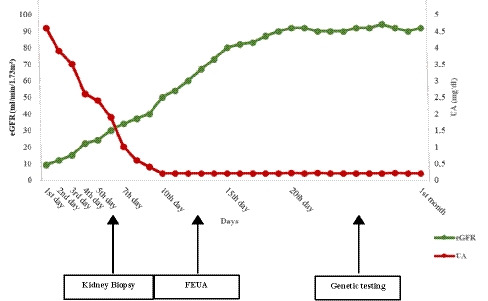
Trend of eGFR (green) and serum acid uric (red) during AKI and in the month following hospital admission. *eGFR* estimated glomerular filtration rate, *UA* serum uric acid, *FEUA* uric acid fractional excretion

A 17-year-old Asian male was admitted to the Emergency Department because of vomiting, fever and bilateral flank pain lasting four days after playing basketball. Familial and previous medical history was unremarkable. Blood pressure and vital parameters were normal. Laboratory tests showed severe kidney function impairment (serum creatinine 8.49 mg/dl), in the absence of acid–base and electrolyte imbalance, and normal UA levels (serum UA 4.6 mg/dl) (Table 1). Urinalysis showed mild microscopic hematuria, mild tubular proteinuria and a pH of 6.0. Immunologic and infectious screenings were negative (Table 1). Kidney ultrasound showed bilateral normal kidney size with mildly increased cortical echogenicity. Urinary tract obstructions, kidney stones, and nephrocalcinosis were ruled out. Despite prompt aggressive hydration, severe kidney injury persisted, and a kidney biopsy was performed. Renal pathology showed signs of acute tubular necrosis, without alterations in the glomerular and vascular compartments. Immunofluorescence staining for C3, C4, immunoglobulins and fibrinogen were all negative (Fig. 1). The patient was treated with supportive therapy and showed progressive improvement of kidney function with complete recovery 14 days after onset (Fig. 2). Of note, as kidney function improved, UA levels progressively decreased, becoming undetectable ten days after hospital admission (Fig. 2). At that time, fractional excretion of UA (FEUA) was 133% (normal values 4–8%), strongly suggesting RHUC. Genetic testing with massive parallel sequencing ((MPS); WES-based virtual gene panel for genes responsible for inherited kidney disorders) showed the presence of a novel pathogenic (class 5) homozygous missense variant c.[361G > A] in the *SLC2A9* gene, thus confirming the clinical suspicion of RHUC. The patient was discharged without sequelae. In the following three years, kidney function remained normal and serum UA was persistently undetectable.

**Table 1:** Laboratory tests at hospital admission

Parameter	Results	Normal value	Parameter	Results	Normal value
Hb	14.5 g/dl	13.5–16.9 g/dl	C3	140 mg/dl	86–160 mg/dl
Red blood cells	4.85 × 10^4^/μL	401–540 × 10^4^/μl	C4	37 mg/dl	17–45 mg/dl
Hematocrit	40%	39.0–51.2%	Antinuclear Ab	< 40 IU	≤ 40 IU
WBC	9570/μl	3100–9500/μl	Anti-dsDNA Ab	Negative	Negative
Platelets	33 × 104/μl	15.1–34.9 × 104/μl	Anti-Sm Ab	Negative	Negative
sUA	4.6 mg/dl	3.7–7.0 mg/dl	ANCA Ab	Negative	Negative
sCr	8.49 mg/dl	0.5–1.1 mg/dl	PCT	< 0.05 ng/ml	< 0.05 ng/ml
eGFR	8 ml/min/1.73 m^2^	≥ 90 mL/min/1.73 m^2^	HIV Ab	Negative	Negative
BUN	90 mg/dl	7.2–50.0 mg/dl	HbsAg	Negative	Negative
CPK	315 IU/l	50–230 IU/l	HCV Ab	Negative	Negative
LDH	290 IU/l	120–245 IU/l	Urinary protein (dipstick)	1+	Negative
Myoglobin	63 μg/ml	≤ 60.0 μg/ml	Glucose	Negative	Negative
Sodium	141 mEq/l	136–145 mEq/l	Hemoglobin	Negative	Negative
Potassium	4 mEq/l	3.6–4.8 mEq/l	Red blood cells	25	< 10/HPF
Chloride	102 mEq/l	99–109 mEq/l	Proteinuria	346.8 mg/24H	< 150 mg/24H
Calcium	9 mg/dl	8.4–10.4 mg/dl	Urinary pH	6.0	5.5–6.5
Magnesium	1.8 mg/dl	1.8–2.4 mg/dl	Urinary UA	996 mg/l	125–300 mg/l
Bicarbonate	22.4 mmol/l	22–24 mmol/l	Und. urinary UA	80 mg/l	< 100 mg/l

*Hb* hemoglobin, *WBC* white blood cells, *sUA* serum uric acid, *sCr* serum creatinine, *eGFR* estimated GFR (calculated by the revised Schwartz formula), *BUN* blood urea nitrogen, *CK* creatine phosphokinase, *LDH* lactic dehydrogenase, *CRP* C-reactive protein, P*C*T procalcitonin, *H* hours, *Ab* antibody, *HPF* high power field, dsDNA double strand DNA, *ANCA* antineutrophil cytoplasmic antibody, *HIV* human immunodeficiency virus, *HCV* hepatitis C virus, *Urine Ph* dipstick, *UA* uric acid, *Und* undissociated (calculated by the Henderson–Hasselbach formula, p*K*a uric acid = 5.4)

## Discussion

This case is emblematic of the issues that come up when considering RHUC in the differential diagnosis of AKI. Currently available guidelines on diagnosis and management of RHUC provide limited support in defining clues for the differential diagnosis of RHUC, which is usually suspected when hypouricemia is found in asymptomatic patients [6]. The lack of indications regarding the appropriate diagnostic workflow when dealing with suspected RHUC-AKI represents an important gap in clinical management. In addition, data about the clinical picture of patients mostly derive from case reports and small case series, preventing clinicians hampering the effort to obtain high-quality evidence that is needed to develop diagnostic strategies. To address these issues, we collected information about all the cases of RHUC-AKI reported in the literature and reviewed clinical, laboratory, genetic and outcome features of the disease (see Supplementary Material for details). We included 40 patients with RHUC and AKI (Table 2). Results of genetic testing were available for 23 patients, all carrying biallelic pathogenic variants in *SCL22A12* or *SCL2A9*. Demographic, clinical and laboratory data did not differ between patients with clinical and genetic diagnosis, except for the evidence of disease relapses during follow-up (Table 2). Most patients (88%) with a clinical diagnosis did not have follow-up data, resulting in a lower number of disease relapses (i.e., episodes of AKI) in comparison to patients with a genetically confirmed diagnosis (Table 2). Most patients were males (90%) and of self-reported Asian ancestry (93%), with a median age at AKI-onset of 18 years (Table 2). Data regarding previous medical history was missing for most patients. A trigger event (exercise, gastroenteritis) preceding the development of AKI was reported in nearly all cases (Table 2). All the patients showed at least one prodromal symptom at clinical presentation, with flank pain and nausea/vomiting representing the most frequently reported ones. Common laboratory data were:

- Severe increase in serum creatinine levels coupled with mildly increased urea levels;
- Absence of electrolyte abnormalities;
- Mild urinary abnormalities;
- Increased FEUA associated with normal UA levels.

**Table 2:** Clinical features of patients with RHUC

	Features	Group with genetic diagnoses (*n* = 23)	Group with clinical diagnosis (*n* = 17)	*P* value	Overall group (*n* = 40)
Demographic data, familial and personal history	Male sex, *n* (%)	21/23 (91%)	15/17 (88.2%)	NS	36/40 (90%)
	Asian ethnicity, *n* (%)	19/22 (86%)	17/17 (100%)	NS	36/39 (93%)
	Age (years), median (IQR)	19 (12.5–24)	19 (12.5–29)	NS	18 (12.5–26)
	Other renal disease, *n* (%)	2/23 (9%)	1/17 (6%)	NS	3/40 (7.5%)
	Family history, *n* (%)				
	Yes	8/23 (35%)	4/17 (23.5%)		12/40 (30%)
	Kidney stones	5/23 (22%)	1/17 (6%)		6/40 (15%)
	AKI	2/23 (9%)	0/17 (0%)	NS	2/40 (5%)
	Hypouricemia	0/23 (0%)	1/17 (6%)		1/40 (2.5%)
	Kidney failure	1/23 (4%)	3/17 (17.5%)		4/40 (10%)
	Previous episodes AKI, *n* (%)	2/23 (9%)	2/17 (12%)	NS	4/40 (10%)
	Absence of previous evaluation of UA, *n* (%)	21/23 (87%)	16/17 (93%)	NS	37/40 (92.5%)
Clinical features	Trigger, *n* (%)				
	No trigger	2/23 (9%)	0/17 (0%)		2/40 (5%)
	1 trigger	20/23 (87%)	17/17 (100%)	NS	37/40 (92.5%)
	> 1 trigger	1/23(4%)	0/17 (0%)		1/40 (2.5%)
	Symptoms and signs, *n* (%)				
	Loin pain	22/23(96%)	17/17 (100%		39/40 (97%)
	Nausea/vomiting	19/23 (83%)	16/17 (97%)		35/40 (87.5%)
	Fever	4/23 (17%)	1/17 (6%)	NS	5/40 (12.5%)
	Hypertension	11/23 (48%)	5/17 (29%)		16/40 (40%)
	Oligo/anuria	8/23 (35%)	3/17 (17.5%)		11/40 (27.5%)
	Number of symptoms, *n* (%)				
	1	3/23 (13%)	2/17(12%)		5/40 (15%)
	2	6/23 (26%)	8/17 (47%)	NS	14/40 (35%)
	3	7/23 (30%)	3/17 (17.5%)		10/40 (25%)
	> 4	7/23 (30%)	4/17 (23.5%)		11/40 (27.5%)
	Extrarenal manifestations, *n* (%)^a^	7/23 (30%)	2/17(12%)	NS	9/40 (22.5%)
Laboratory findings	sCr (mg/dl), median (IQR)	3.5 (2.75–4.25)	4.89 (2.75–6)	NS	4.7 (2.75–5.75)
	eGFR (ml/min/1.73), median (IQR)	15 (8.5–30.5)	12 (8.5–30.5)	NS	13 (9–23.5)
	Urea (mg/dl), median (IQR)	55 (33–66-5)	49 (33–55.5)	NS	50 (29.6–60)
	UA (mg/dl), median (IQR)	3.9 (1–4)	4 (1–4.25)	NS	4 (2–4.25)
	Calcium (mg/dl), median (IQR)	8.5 (8–8.8)	8.5 (8–8.8)	NS	8.5 (8–8.8)
	Phosphate (mg/dl), median (IQR)	4 (3.9–4.2)	4.3 (4–4.7)	NS	4 (4–4.6)
	Potassium (mmol/l), median (IQR)	4.2 (3.7–4.7)	4.2 (3.8–4.3)	NS	4 (3.7–4.4)
	Sodium (mmol/l), median (IQR)	141 (136–142)	142 (136–142.5)	NS	141.3 (137–141)
	Proteinuria at dipstick, *n* (%)	9/20 (47%)	9/17 (52%)	NS	18/37 (48%)
	Hematuria, *n* (%)	6/22 (27%)	3/17 (17%)	NS	9/39 (23%)
	CPK (UI/ml), median (IQR)	267 (118–572)	170 (117–298)	NS	223 (117–392)
	FEUA (%), median (IQR)	77 (52–135)	56 (50–69)	NS	66 (49–127)
Clinical management	Kidney biopsy, *n* (%)	9/23 (40%)	7/17 (41%)	NS	16/40 (40%)
	Dialysis, *n* (%)	6/23 (26%)	3/17 (17.6%)	NS	9/40 (22.5%)
	Length of hospitalization (days), median (IQR)	14 (10.75–19)	14 (10–18)	NS	14 (10–18.5)
AKI Relapse	NA, *n* (%)	10/23 (43%)	15/17 (88%)	< 0.05	25/40 (62.5%)
	Yes, *n* (%)	8/23 (35%)	1/17 (6%)	< 0.05	9/40 (22.5%)
Genetic results	*SCL2A9*, *n* (%)	11/23 (48%)	NA	NA	NA
	*SCL22A12*, *n* (%)	12/23 (52%)	NA	NA	NA

*n* number, *IQR* interquartile range, *AKI* acute kidney injury, *UA* uric acid, *sCr* serum creatinine, *eGFR* estimated glomerular filtration rate, *CPK* creatine phosphokinase, *FEUA* fractional excretion of uric acid, *SCL2A9 Solute carrier family* 2 facilitated glucose transporter member 9, *SCL22A12 solute* Carrier Family 22 Member 12, *NA* not available, *NS* not statistically significant

^a^Extrarenal manifestations: cardiovascular involvement (i.e., acute pulmonary edema), neurological involvement (i.e., posterior reversible encephalopathy), gastrointestinal involvement (i.e., hyperbilirubinemia)

Of note, uric acid  in the acute phase of AKI is the norm in the majority of patients, likely due to the rise in muscle release and the decline in kidney function [7]. The estimated glomerular filtration rate (eGFR) and uric acid frequently show an opposite trend during the disease course, with eGFR increasing over time while uric acid, in the normal range at onset, becomes undetectable [8, 9], as can be observed in our patient (Fig. 2). These data support the hypothesis that the only parameter suggesting abnormalities in uric acid handling and a diagnosis of RHUC in patients with RHUC-AKI is FEUA, which is rarely measured as a first-line investigation in the diagnostic work-up of patients with AKI. Nonetheless, FEUA represents an inexpensive, non-invasive diagnostic tool to address clinical suspicion in patients with clinical and laboratory “red flags”.

In patients with RHUC-AKI, genetic testing should be considered in order to obtain a conclusive diagnosis and to assess genotype–phenotype correlations [6]. According to local availability and confidence in interpreting the results, direct genotyping with Sanger sequencing can even be considered as an early-option diagnostic tool to provide rapid genetic results and a definitive diagnosis of the disease. Of note, Sanger sequencing failed to identify pathogenic variants in three patients (8%) with a clinical picture of RHUC-AKI included in our cohort analysis [10, 11]. In the future, MPS could be considered in order to identify additional genes involved in the pathogenesis of RHUC and to perform reliable genotype–phenotype correlations. Of note, a familial history of hypouricemia, AKI, kidney stones and kidney failure was reported in 2.5%, 5%, 15% and 10% of the patients, respectively. The lack of data concerning the genotype of the relatives hampered the assessment of a correlation between heterozygous variants in disease-causing genes and the clinical manifestations. Since the possibility of a clinical phenotype has been reported also for carriers of variants in the *SLC22A2* and *SLC2A9* genes [2, 3], unraveling the relationship between genotype and clinical manifestations would be relevant for understanding the pathophysiology of kidney damage in patients with hypouricemia.

The importance of genetic testing is reinforced by the observation that a conclusive genetic diagnosis of RHUC results in changes in follow up. Given the usual spontaneous recovery of kidney function without apparent sequelae (77.5% of patients recover with supportive therapy), the clinical management of patients with RHUC-AKI could consist in a “wait-and-see” monitoring approach, supportive therapy and rapid detection of AKI complications. Follow-up data are reported in only 37.5% of patients, who relapsed in 40% of cases. Interestingly, all relapses occurred in patients with a genetically confirmed diagnosis, while the majority of patients who received a clinical diagnosis of RHUC did not have follow-up data, resulting in a lower number of disease relapses. These data could possibly be explained by the nephrologist’s awareness of the disease resulting from positive genetic testing and the consequent follow-up, rather than to a more aggressive phenotype. This observation suggests that genetic confirmation of RHUC can help to set up an appropriate long-term follow-up in these patients, preventing further relapses and avoiding additional kidney injury.

Pathomechanisms responsible for AKI in RHUC are not fully elucidated. Intratubular precipitation of UA causing urate nephropathy was one of the first suggested hypotheses [1, 12]. Kidney biopsy in patients with urate nephropathy usually shows crystalline shaped spaces representing urate crystals that dissolved after tissue processing. Crystals are usually surrounded by an intense inflammatory infiltrate [1, 12]. These aspects have not been reported in patients with RHUC-AKI, making this hypothesis unlikely. Moreover, crystal precipitation is pH-dependent, occurring when urinary pH falls below 6.0, thus resulting in ultrasound supersaturation. In our patient, the concentration of undissociated UA was below the solubility limit of 100 mg/l both at AKI onset (80 mg/l) and at the last follow-up (62 mg/l). This probably also explains the unexpectedly low prevalence of kidney stones in patients with RHUC-AKI (Table 2).

Previous studies reported on a direct action of high intratubular uric acid concentrations on the activation of the toll-like receptor 4 (TLR4) pathway and the nucleotide binding oligomerization domain-like receptor family pyrin domain-containing 3 (NLRP3) inflammasome, leading to an increased release of interleukin-1β (IL-1β) [1, 13]. IL-1β can induce the activation of the sympathetic nerve, that in turn constricts afferent arterioles causing a decrease of GFR and AKI, and the activation of the intrarenal sensory nerve causing lumbar pain, which is among the most common presenting symptoms in our cohort [1].

Finally, a recent paper suggested that RHUC-AKI is caused by an abnormal salvage pathway of purines during anaerobic exercise [14]. RHUC-AKI patients show an altered capability to synthesize adenosine triphosphate (ATP) from hypoxanthine, which is lost at a high rate in urine. The authors suggest that the lack of ATP in tubules can lead to higher susceptibility of stress-induced hypoxia and exercise-induced kidney damage.

Interestingly, all patients with a clinical picture of RHUC-AKI who underwent genetic testing showed biallelic pathogenic variants in *SLC2A9* or *SLC22A12*, while carriers were not identified. Isolated hypouricemia is also described in heterozygous patients [2, 3], albeit more rarely than in homozygous ones[15]. These observations suggest that, although hypouricemia is associated with a risk of developing AKI in patients with RHUC, it is most likely unable to cause kidney injury per se. In this view, patients with RHUC frequently show a trigger event preceding AKI, which can therefore result from the combination of hypouricemia with additional hits (e.g., dehydration) to a specific genetic background. It is reasonable to hypothesize that the loss of functional copies of the transporter proteins due to biallelic variants can result in greater susceptibility to additional insults, although this needs to be experimentally proven.

Long-term data on kidney prognosis in patients with RHUC are lacking. As a matter of fact, RHUC-AKI can relapse and indeed, in our cohort, 60% of patients with available follow-up data showed at least one relapse. Interestingly, previous studies reported that hypouricemic patients have a ninefold higher rate of prior kidney disease (although clinically unaddressed and reported as nephritis/nephrosis) compared to those without hypouricemia [16]. As widely reported, repeated AKI may lead to progressive decline in eGFR and to CKD [17], and RHUC-AKI is likely no exception. The risk of CKD in hypouricemic patients is further supported by the U-shaped curve that relates uric acid  to eGFR, with kidney function being modulated by either high or low serum uric acid levels [18, 19]. According to the information provided by our cohort and our own observations, we can hypothesize that the latter group contains patients with RHUC developing relapsing AKI leading to kidney function decline over time. Of note, the relationship linking hypouricemia to CKD is observed only in males [18, 19]. Interestingly, most patients with RHUC and AKI are males. Tubular handling of urate differs between males and females, being influenced by sex hormones [20]. Mechanisms for male gender selectivity of RHUC-AKI are not known and further studies are needed to clarify the difference in tubular handling of uric acid in determining the risk of AKI.

In conclusion, our study on RHUC-AKI suggests that: (1) Clinical (e.g., Asian ancestry, male gender, prodromal symptoms preceding AKI) and laboratory findings (e.g., abnormally elevated FEUA) should lead us to suspect RHUC in patients with AKI; (2) Clinical evaluation is reliable in assessing the diagnosis of RHUC and can help avoid kidney biopsy in favor of genetic testing, which is pivotal for assessing genotype–phenotype correlations; (3) RHUC is a risk factor for AKI only in patents with biallelic genetic variants in disease-causing genes; (4) Hypouricemia is not a risk factor for kidney injury per se, and the combination of multiple pathomechanisms such as gender, genetic background and triggers are probably needed to determine AKI and CKD. 

### Supplementary Information

Below is the link to the electronic supplementary material.

Supplementary file1 (DOCX 20 KB)
