# Correction to: Clinical features suggesting renal hypouricemia as the cause of acute kidney injury: a case report and review of the literature

**DOI:** 10.1007/s40620-024-01891-1

**Published:** 2024-01-18

**Authors:** Tommaso Mazzierli, Luigi Cirillo, Viviana Palazzo, Fiammetta Ravaglia, Francesca Becherucci

**Affiliations:** 1 Department of Biomedical, Experimental and Clinical Sciences “Mario Serio”, University of Florence, Florence, Italy; 2 Nephrology and Dialysis Unit, Meyer Children’s Hospital IRCCS, Florence, Italy; 3 Medical Genetics Unit, Meyer Children’s Hospital IRCCS, Florence, Italy; 4 Nephrology and Dialysis Unit, Santo Stefano Hospital, Prato, Italy

**Correction to: Journal of Nephrology (2023) 36:651–657** 10.1007/s40620-022-01494-8

After publication online, the affiliations 2 and 3 were incorrectly. The correction affiliations are copied below.

2 Nephrology and Dialysis Unit, Meyer Children's Hospital IRCCS, Florence, Italy

3 Medical Genetics Unit, Meyer Children's Hospital IRCCS, Florence, Italy

The original article has been corrected.

