# Supplementary material for: Clinical features suggesting renal hypouricemia as the cause of acute kidney injury: a case report and review of the literature
Source: J Nephrol. 2022 Nov 23;36(3):651–7. doi: 10.1007/s40620-022-01494-8 (PMC10089983; doi:10.1007/s40620-022-01494-8)
Supplement: Supplementary file 1 — Supplementary file1 (DOCX 20 KB) [file 40620_2022_1494_MOESM1_ESM.docx]

**Supplementary Material**

**Methods**

We conducted a PubMed literature search to identify publications reporting AKI in patients with RHUC using the medical subject heading terms “RHUC acute kidney injury”, “renal hypouricemia kidney injury”, “exercise induced kidney injury”. We collected data from 1974, when RHUC was first identified, to May 2022 [1-35]. We excluded case reports with incomplete or unavailable clinical data. We included both clinically suspected RHUC and RHUC confirmed by the genetic testing. For each patient, we collected demographic, clinical and laboratory data including:

- Presence/absence of triggers preceding AKI;
- Prodromal symptoms: loin pain, fever, nausea/vomiting;
- Blood tests at onset of AKI: serum levels of creatinine, UA, electrolytes, creatine phosphokinase and myoglobin;
- Urinary findings at diagnosis of AKI: proteinuria (urine dipstick), hematuria (urine dipstick) , FEUA;
- Results of instrumental examinations: kidney ultrasound or CT scan;
- Therapy: supportive therapy and/or kidney replacement therapy (KRT);
- Length of hospitalization;
- Results of genetic testing, if performed;
- Relapse and follow up data.

Estimated glomerular filtration rate (eGFR) was calculated with revised Schwartz or CKD-EPI formula owing to the age of patients. Data were reported as frequency for categorical variables and median and interquartile range (IQR) for non-categorical variables. Statistical analysis was performed using the R software [36]. We compared groups using Mann-Whitney U test for unpaired data. Data distribution was assessed by Shapiro-Wilk normality test.

**Supplementary Bibliography**

1. Simkin PA, Skeith MD, Healey LA (1974) Suppression of uric acid secretion in a patient with renal hypouricemia. Adv Exp Med Biol 41:723-8.

2. [Yim J, Kim M, Suh J-S (2022) Hereditary renal hypouricemia with SLC22A12 mutation: A case report. Pediatr Neonatol 63:202–203](http://paperpile.com/b/u8UQSc/hcp3)

3. [Kaynar K, Güvercin B, Şahin M, et al (2021) A novel mutation in a patient with familial renal hypouricemia type 2. Nefrologia](http://paperpile.com/b/u8UQSc/9D8p) 16:S0211-6995(21)00154-5

4. [Maalouli C, Dahan K, Devresse A, Gillion V (2021) Mutation in the Gene: A New Family with Familial Renal Hypouricemia Type 2. Case Rep Nephrol Urol 2021:4751099](http://paperpile.com/b/u8UQSc/CXux)

5. [Jeannin G, Chiarelli N, Gaggiotti M, et al (2014) Recurrent exercise-induced acute renal failure in a young Pakistani man with severe renal hypouricemia and SLC2A9 compound heterozygosity. BMC medical genetics 15:3](about:blank)

6. [Shimizu Y, Wakabayashi K, Totsuka A, et al (2019) Exercise-Induced Acute Kidney Injury in a Police Officer with Hereditary Renal Hypouricemia. Case Rep Nephrol Dial 9:92–101](http://paperpile.com/b/u8UQSc/5rhB)

7. [Wang C, Wang J, Liu S, et al (2019) Idiopathic renal hypouricemia: A case report and literature review. Mol Med Rep 20:5118–5124](http://paperpile.com/b/u8UQSc/gHWx)

8. [Windpessl M, Ritelli M, Wallner M, Colombi M (2016) A Novel Homozygous SLC2A9 Mutation Associated with Renal-Induced Hypouricemia. Am J Nephrol 43:245–250](http://paperpile.com/b/u8UQSc/YmbJ)

9. [Fujinaga S, Nishino T (2016) Acute kidney injury due to rotavirus gastroenteritis-associated obstructive uric acid stones in a Japanese infant harboring a heterozygous W258X mutation. Pediatr. Nephrol 31:2379–2380](http://paperpile.com/b/u8UQSc/J5jn)

10. [Kim HO, Ihm C-G, Jeong KH, et al (2015) A Case Report of Familial Renal Hypouricemia Confirmed by Genotyping of SLC22A12, and a Literature Review. Electrolyte Blood Press 13:52–57](http://paperpile.com/b/u8UQSc/OH45)

11. [Mou L-J, Jiang L-P, Hu Y (2015) A novel homozygous GLUT9 mutation cause recurrent exercise-induced acute renal failure and posterior reversible encephalopathy syndrome. J Nephrol 28:387–392](http://paperpile.com/b/u8UQSc/ve2o)

12. [Shen H, Feng C, Jin X, et al (2014) Recurrent exercise-induced acute kidney injury by idiopathic renal hypouricemia with a novel mutation in the SLC2A9 gene and literature review. BMC Pediatr 14:73](http://paperpile.com/b/u8UQSc/A08B)

13. [Fujinaga S, Ito A, Nakagawa M, et al (2013) Posterior reversible encephalopathy syndrome with exercise-induced acute kidney injury in renal hypouricemia type 1. Eur J Pediatr 172:1557–1560](http://paperpile.com/b/u8UQSc/YV6T)

14. [Chakraborty S, Sural S (2013) A young patient of hereditary renal hypouricaemia presenting with exercise-induced rhabdomyolysis and acute kidney injury. Ann Clin Biochem 50:271–273](http://paperpile.com/b/u8UQSc/43fT)

15. [Li Z, Ding H, Chen C, et al (2013) Novel URAT1 mutations caused acute renal failure after exercise in two Chinese families with renal hypouricemia. Gene 512:97–101](http://paperpile.com/b/u8UQSc/6q90)

16. [Hirashio S, Yamada K, Naito T, Masaki T (2012) A case of renal hypouricemia and a G774A gene mutation causing acute renal injury that was improved by hemodialysis. CEN Case Rep 1:24–28](http://paperpile.com/b/u8UQSc/PuR0)

17. [Ochi A, Takei T, Ichikawa A, et al (2012) A case of acute renal failure after exercise with renal hypouricemia demonstrated compound heterozygous mutations of uric acid transporter 1. Clin Exp Nephrol 16:316–319](http://paperpile.com/b/u8UQSc/O14S)

18. [Stiburkova B, Taylor J, Marinaki AM, Sebesta I (2012) Acute kidney injury in two children caused by renal hypouricaemia type 2. Pediatr Nephrol 27:1411–1415](http://paperpile.com/b/u8UQSc/h7g4)

19. [Kim YH, Cho JT (2011) A case of exercise-induced acute renal failure with G774A mutation in SCL22A12 causing renal hypouricemia. J Korean Med Sci 26:1238–1240](http://paperpile.com/b/u8UQSc/eMAF)

20. [Shima Y, Nozu K, Nozu Y, et al (2011) Recurrent EIARF and PRES with severe renal hypouricemia by compound heterozygous SLC2A9 mutation. Pediatrics 127:e1621–5](http://paperpile.com/b/u8UQSc/JYDL)

21. [Yan M-T, Cheng C-J, Chen J-S, Lin S-H (2010) The case: a young man with acute kidney injury after exercise. The diagnosis: exercise induced acute kidney injury in hereditary renal hypouricemia. Kidney Int 77:935–936](http://paperpile.com/b/u8UQSc/79xT)

22. [Mima A, Ichida K, Matsubara T, et al (2008) Acute renal failure after exercise in a Japanese sumo wrestler with renal hypouricemia. Am J Med Sci 336:512–514](http://paperpile.com/b/u8UQSc/hYQe)

23. [Ishikawa I (2008) Exercise-Induced Acute Renal Failure: Acute Renal Failure with Severe Loin Pain and Patchy Renal Ischemia after Anaerobic Exercise.](http://paperpile.com/b/u8UQSc/A4ds) Nephron 91:559-70

24. [Sugimoto T, Ide R, Uzu T, Kashiwagi A (2007) Recurring exercise-induced acute renal failure with usual daily work. Nephrology 12:110](http://paperpile.com/b/u8UQSc/JMdR)

25. [Nishida H, Kaida H, Ishibashi M, et al (2005) Evaluation of exercise-induced acute renal failure in renal hypouricemia using Tc-99m DTPA renography. Ann Nucl Med 19:325–329](http://paperpile.com/b/u8UQSc/doSn)

26. [Tanaka M, Itoh K, Matsushita K, et al (2003) Two male siblings with hereditary renal hypouricemia and exercise-induced ARF. Am J Kidney Dis 42:1287–1292](http://paperpile.com/b/u8UQSc/OxYq)

27. [Ito O, Hasegawa Y, Sato K, et al (2003) A case of exercise-induced acute renal failure in a patient with idiopathic renal hypouricemia developed during antihypertensive therapy with losartan and trichlormethiazide. Hypertens Res 26:509–513](http://paperpile.com/b/u8UQSc/Yaa7)

28. [Watanabe T, Abe T, Oda Y (2000) Exercise-induced acute renal failure in a patient with renal hypouricemia. Pediatr Nephrol 14:851–852](http://paperpile.com/b/WWWi04/dK7Y)

29. [Kurihara I, Soma J, Sato H, et al (2000) A case of exercise-induced acute renal failure in a patient with enhanced renal hypouricaemia. Nephrology Dialysis Transplantation 15:104–106](http://paperpile.com/b/u8UQSc/cypD)

30. [Sato T, Kuno T, Tashiro K, et al (1998) Exercise-induced acute renal failure in a girl with renal hypouricemia. Acta Paediatr Jpn 40:93–95](http://paperpile.com/b/WWWi04/tS1V)

31. [Ninomiya M, Ito Y, Nishi A, et al (1996) Recurrent exercise-induced acute renal failure in renal hypouricemia. Acta Paediatr 85:1009–1011](http://paperpile.com/b/u8UQSc/aRQJ)

32. [Yeun JY, Hasbargen JA (1995) Renal hypouricemia: prevention of exercise-induced acute renal failure and a review of the literature. Am J Kidney Dis 25:937–946](http://paperpile.com/b/u8UQSc/72by)

33. [Fujieda M, Yokoyama W, Oishi N, et al (1995) Acute renal failure after exercise in a child with renal hypouricemia. Acta Paediatr Jpn 37:642–644](http://paperpile.com/b/u8UQSc/bGQX)

34. [Igarashi T, Sekine T, Sugimura H, et al (1993) Acute renal failure after exercise in a child with renal hypouricaemia. Pediatr Nephrol 7:292–293](http://paperpile.com/b/u8UQSc/w0P3)

35. [Ishikawa I, Sakurai Y, Masuzaki S, et al (1990) Exercise-induced acute renal failure in 3 patients with renal hypouricemia. Nihon Jinzo Gakkai Shi 32:923–928](http://paperpile.com/b/u8UQSc/FwuM)

36. R Core Team (2021). R: A language and environment for statistical computing. R Foundation for Statistical Computing, Vienna, Austria. [https://www.R-project.org/](https://www.r-project.org/).
